# LGR5 Is a Gastric Cancer Stem Cell Marker Associated with Stemness and the EMT Signature Genes *NANOG*, *NANOGP8*, *PRRX1*, *TWIST1*, and *BMI1*

**DOI:** 10.1371/journal.pone.0168904

**Published:** 2016-12-29

**Authors:** Bei Wang, Queting Chen, Yang Cao, Xia Ma, Chenxing Yin, Youchao Jia, Aimin Zang, Wufang Fan

**Affiliations:** 1 Molecular Biology Lab of Gastric Cancer, School of Life Sciences, Hebei University, Baoding, Hebei Province, China; 2 Department of Medical Oncology, Affiliated Hospital of Hebei University, Baoding, Hebei Province, China; Second University of Naples, ITALY

## Abstract

**Background:**

Accumulating evidence supports the hypothesis that cancer stem cells (CSCs) are essential for cancer initiation, metastasis and drug resistance. However, the functional association of gastric CSC markers with stemness and epithelial-mesenchymal transition (EMT) signature genes is unclear.

**Methods:**

qPCR was performed to measure the expression profiles of stemness and EMT signature genes and their association with putative CSC markers in gastric cancer tissues, cancer cell lines and sphere cells. Western blot analysis was used to confirm the results of the transcript analysis. Cell proliferation, cell migration, drug resistance and sphere cell growth assays were conducted to measure the expansion and invasion abilities of the cells. Tumor xenograft experiments were performed in NOD/SCID mice to test cell stemness *in vivo*. Flow cytometry and immunofluorescence staining were used to analyze cell subpopulations.

**Results:**

The expression of *LGR5* was strikingly up-regulated in sphere cells but not in cancer tissues or parental adherent cells. The up-regulation of *LGR5* was also positively associated with stemness regulators (*NANOG*, *OCT4*, *SOX2*, and *AICDA*) and EMT inducers (*PRRX1*, *TWIST1*, and *BMI1)*. In addition, sphere cells exhibited up-regulated vimentin and down-regulated E-cadherin expression. Using gene-specific primers, we found that the *NANOG* expression primarily originates from the retrogene *NANOGP8*. Western blot analysis showed that the expression of both LGR5 and NANOG is significantly higher in sphere cells. *LGR5* over-expression significantly enhanced sphere cell growth, cell proliferation, cell migration and drug resistance in MGC803 cells. Tumor xenografts in nude mice showed that sphere cells are at least 10 times more efficient at tumor initiation than adherent cells. Flow cytometry analysis showed that ~20% of sphere cells are LGR5+/CD54+, but only ~3% of adherent cells are Lgr5+/CD54+. Immunofluorescence staining supports the above results.

**Conclusion:**

The *LGR5*-expressing fraction of CD54+ cells represents gastric cancer CSCs, in which *LGR5* is closely associated with stemness and EMT core genes, and *NANOG* expression is mainly contributed by the retrogene *NANOGP8*. Sphere cells are the best starting materials for the characterization of CSCs.

## Introduction

Gastric cancer (GC) is a typical epithelium-originated malignant tumor. It is the second most common cancer worldwide and the second most common cause of cancer-related deaths [1]. With nearly one million new cases diagnosed yearly and more than 700,000 GC-related deaths per year, GC poses a significant public health problem around the globe. A comprehensive understanding of the molecular etiopathogenesis of GC has lagged behind many other cancers because of the lack of knowledge for identifying the genetic risk of susceptibility and somatic drivers of cancer progression. The recent cancer stem cell (CSC) hypothesis proposes that only a small fraction of cancer stem cells is responsible for self-renewal and differentiation into heterogeneous cancer cells. In fact, CSCs have been isolated from many solid cancers, such as glioblastoma, melanoma, prostate carcinoma, colon carcinoma, head and neck squamous cell carcinoma, breast carcinoma, ovarian carcinoma, bladder carcinoma, lung carcinoma, and pancreatic carcinoma [2].

It has been reported that the aberrant expression of stemness factors drives CSC initiation and establishment [3, 4, 5]. Increasing evidence shows that CSCs can potentially arise from oncogenic reprogramming of normal stem cells, in which the essential transcription factors for stemness, such as NANOG, OCT4 and SOX2, play an indispensable role. Many studies have demonstrated that CSCs are a group of cells with characteristics of both stemness and EMT [6]. Some data suggest that CSCs arising from epithelial tissues generally express a mixture of epithelial and mesenchymal features, indicating that the mechanisms modulating stemness and EMT are closely coupled together [7, 8]. If this is the case, a given CSC marker should be intimately associated with both stemness and EMT regulators.

LGR5 has been reported to be a biomarker for both adult stem cells and CSCs in the gastrointestinal tract [9,4,10] in mice, and its expression is correlated with other putative CSC markers such as Bmi1 [11]. Several groups have reported different proteins as gastric CSC markers, such as CD44+ [12], ALDH1+ [13], CD44+/CD54+ [14], CD44/CD24+ cells [15], but so far none of these have been confirmed to be a functional CSC marker. In fact, few reports have presented evidence regarding the association of these so-called CSC markers with cell stemness and EMT properties.

A definitive demonstration of CSC characteristics first requires the isolation of CSCs. Many methods have been employed for isolating these cells. In the simplest method, a marker is chosen to allow for the separation of the marker-specific sub-population from a given cancer tissue or cell line by flow cytometry, the sorted cells are inoculated into nude mice, and the readout is the tumor-initiation capacity of the cells [16, 17]. The key questions are how to choose these markers prior to sorting the CSCs and how to distinguish functional markers from passenger markers that play no physiological role in stemness and EMT properties. Considering the very tiny number of CSCs hidden in the overwhelming bulk of the tumor mass, there is frequently too much noise to detect the signal emitted by CSCs. Therefore, the most logical procedure is to enrich for the stem-like cells first, and then identify the *bona fide* CSC marker(s). Currently, there are two approaches to isolate stem-like cells independent of markers, i.e., sphere cell culture [16, 18] and side-population isolation [19, 20]. Many studies have demonstrated that sphere cell culture is a practical way to obtain CSC-like cells from solid tumors [21, 22], but using this method to analyze the stemness and EMT properties of gastric CSCs has not yet been reported.

The aim of this study is to assess (1) the usefulness of cancer tissues, cancer cell lines and sphere cells in the characterization of CSCs; (2) whether the stemness and EMT properties are coupled together in sphere cells (CSC-like cells); (3) which CSC marker is closely associated with stemness and EMT properties in gastric cancer cells; and (4) the tumor cell biology properties that the CSC-like cells demonstrate. Here, we present the data.

## Materials and Methods

### Subjects and tissue samples

Paired tissue samples were collected from 9 gastric cancer patients who underwent a gastrectomy procedure during 2014 at the Affiliated Hospital of Hebei University (Baoding). The adjacent normal gastric tissues were collected at least 5 cm away from the carcinoma. The fresh tissues samples were frozen in liquid nitrogen until they were used for total RNA extraction. The study was conducted in the cancer research laboratory of Hebei University, Baoding. The hospital institutional ethical review committee (Ethical Review Committee of Affiliated Hospital of Hebei University) approved this study protocol, and all patients provided written informed consent.

### Cell lines and sphere culture

The human gastric adenocarcinoma cell lines MGC803 (3111C0001CCC000227), MKN45 (3111C0001CCC000229), SGC-7901 (3111C0001CCC000236), and HGC27 (3111C0001CCC000279) were purchased from the Institute of Basic Medical Sciences of the Chinese Academy of Medical Sciences (Beijing, China), and the human gastric epithelial cell line GES-1 [23] was purchased from the Laboratory of Genetics at Beijing Cancer Hospital (Beijing, China). All the cell lines were maintained in high glucose DMEM with 10% fetal bovine serum (FBS), 100 IU/ml penicillin G and 100 μg/ml streptomycin at 37°C in a humidified 5% CO2 incubator. For sphere formation, cells were collected, washed, suspended in serum-free DMEM containing 1% N-2 (17502–048, Gibco, USA) and 2% B-27 supplements (17504–044, Gibco, USA), 100 U of a penicillin/streptomycin mixture (Shijiazhuang Pharmaceutical Group Co., Ltd.), 20 ng/ml human Fibroblast Growth Factor-basic (bFGF, FGF-2) (GF003, Millipore, Temecula, CA, USA) and 100 ng/ml Epidermal Growth Factor-basic (EGF) (GF144, Millipore, Temecula, CA, USA) and subsequently cultured in ultra-low attachment 6-well plates (Corning Inc., Corning, NY, USA) at a density of approximately 5,000 cells per well for 14 days per generation.

### qPCR and primers

Total RNA was extracted from the parental cells and sphere-forming cells using RNAiso Plus (Takara Bio Inc., Japan) according to the instructions. Reverse transcription reactions to transcribe 2 μg of total RNA into cDNA were performed with TransScript One-Step gDNA Removal and cDNA Synthesis Super Mix (Transgen Biotech, China). To determine the fold change in the expression of each gene, real-time qPCR was performed using a SYBR Premix Ex TaqII PCR kit (Takara Bio Inc., Japan) in an Applied Biosystems 7500 Real-Time PCR System. The reaction mixture of 20 μl contained 10 μl of SYBR Premix Ex TaqII PCR mix (Takara Bio Inc., Japan), 2 μl of primers (10 mM) and 8 μl of template cDNA (0.4 μg). The *GAPDH* gene served as an internal control. The primer sequences are summarized in Table 1. After an initial incubation for 3 min at 95°C, the reactions were carried out for 39 cycles at 95°C for 20 sec and 60°C for 30 sec (fluorescence collection). Reactions with no template were included as a negative control. By setting the threshold at a level corresponding to the middle of the linear phase of the amplification curve, the Ct values of the target genes were calculated using the 7500 system SDS 1.4 software, and the 2(-ΔΔC(T)) method was used. For each sample, the qPCRs were performed in triplicate for three times with each pair of primers.

**Table 1:** Primers used in this study.

Gene	Primers
LGR5	5'-TCTGGTGAGCCTGAGAAAGC-3'
	5'-ATGCTGGAGCTGGTAAAGGT-3'
CD44	5'-AGAGGCTGAGACAGGAGGTT-3'
	5'-GCTTCCAGAGTTACGCCCTT-3'
ALDH1	5'-TTTGTCCAGCCCACAGTGTT-3'
	5'-ACGCCATAGCAATTCACCCA-3'
CD24	5'-CAGATCCAAGCATCCTGAGCA-3'
	5'-CGTGGTCAATGCAATTCTACTCT-3'
CD54	5'-ACACTAGGCCACGCATCTG-3'
	5'-TCATGGTGGGGCTATGTCTC-3'
BMI1	5'-TTGTTGCAGTGAAGAAAAACCT-3'
	5'-TTCAGACATAGCAGAAGGCA-3'
TWIST1	5'-GCCGGAGACCTAGATGTCATT-3'
	5'-CCCACGCCCTGTTTCTTTGA-3'
PRRX1	5'-TGGAGCTTGAAGAGAATGGCT-3'
	5'-TTCAGGCTTTGCTGTTTGCC-3'
NANOG	5'-TCTGGACACTGGCTGAATCC-3'
	5'-TGACTGGATGGGCATCATGG-3'
OCT4	5'-AGGTATTCAGCCAAACGACCA-3'
	5'-GCACGAGGGTTTCTGCTTTG-3'
AID	5'-TGCTTGAATGTTGGGGAGAGG-3'
	5'-GGGAGAAGCATCACACACATACA-3'
SOX2	5'-TACAGCATGATGCAGGACCA-3'
	5'-CGAGCTGGTCATGGAGTTGTA-3'
NANOG1-S	5'-TTCATTATAAATCTAGAGACTCCAGGA-3'
	5'-CTTTGGGACTGGTGGAAGAATC-3'
NANOG1/P8	5'-GCAGAGAAGAGTGTCG-3'
	5'-AGCTGGGTGGAAGAGAACACAG-3'
ECAD	5'-TGTAACTTGCAATGGGCAGC-3'
	5'-CAAGCTCTCCTGCCATCTCC-3'
VIM	5'-ACGTCTTGACCTTGAACGCA-3'
	5'-TCTTGGCAGCCACACTTTCA-3'
GAPDH	5'-AAGAAGGTGGTGAAGCAGGC-3'
	5'-GTCAAAGGTGGAGGAGTGGG-3'

### Protein extraction and western blotting

The total proteins from MGC803 cells and MGC803 sphere cells were extracted with a lysis buffer. The concentration of proteins in the supernatant was analyzed using the BCA method (Beyotime, China). The protein samples (60 μg per lane) were loaded onto 10% SDS-polyacrylamide gels, electrophoresed, and then transferred to nitrocellulose membranes that were first blocked with 10% (wv^-1^) non-fat milk in TBST [50 mM Tris-HCl (pH 7.6), 150 mM NaCl, 0.1% Tween-20] at room temperature for 1 h and then incubated with a primary antibody diluted in TBST (anti-LGR5 mouse mAb, 1:2000, OriGene; anti-beta-actin mAb, 1:5000, Proteintech) overnight at 4°C. After washing three times with TBST, the membranes were incubated with a peroxidase-conjugated Affinipure Goat Anti-Mouse IgG, 1:5000, Proteintech) for 1.5 h at room temperature. The bands were visualized and quantified using the ECL chemiluminescence detection system (FluorChem E, Alpha, USA).

### LGR5 transfection in MGC803 cells

A plasmid encoding C-terminal green fluorescent protein (GFP)-tagged LGR5 (pReceiver-M45-LGR5, LGR5) and a negative control plasmid without LGR5 (pReceiver-M45-NC, mock) were purchased from GeneCopoeia (GeneCopoeia Inc, China). A total of 5x10^5^ MGC803 cells were seeded into each well of a 6-well plate. When the cells reached 80–90% confluency, the MGC803 cells were transfected with 2500 ng of the LGR5 plasmid or mock plasmid and 5 μl of Lipofectamine 3000 (Invitrogen, Carlsbad, CA) according to the manufacturer’s instructions. The transfected cells were harvested after 24 hours for cell proliferation and sphere cell growth assays and 48 hours for western blot analysis.

### Cell proliferation assay

A CCK-8 Cell Counting Kit (Vazyme, Nanjing, China) was used to measure the cell proliferation. After transfection, cells were seeded at a density of 2500 cells per well in 96-well microplates. The cell proliferation was examined after 1, 2, 3, 4, and 5 days. At each time point, 10 μl of CCK8 solution was added to each well and incubated for another 2 h at 37°C. The optical density was detected at a wavelength of 450 nm using a microplate reader (SpectraMax M4, Molecular Devices, USA). Each sample had three replicates.

### Sphere cell growth assay

A sphere formation assay was conducted to assess the stemness properties of LGR5-expressing cells. After 24 hours of LGR5 transfection, the cells were cultured in ultra-low attachment 6-well plates (Corning, USA) at a density of 5000 cells per well in sphere formation medium (DMEM containing 1% N-2 and 2% B-27 supplements, 100 U penicillin/streptomycin mixture, 20 ng/ml bFGF and 100 ng/ml EGF). After 14 days, the number of spheres was counted.

### Cell migration test by a wound-healing assay

MGC803 cells were cultivated in six-well culture plates (Corning Inc., Corning, NY, USA) at a density of 3×10^5^ cells/well and grown to full confluence overnight. Forty-eight hours after transfection, the confluent monolayer cells were scratched by a plastic tip and washed with PBS buffer to remove the cell debris. Aliquots of 0.5% FBS-containing DMEM were then added to each well, and the scratched monolayer was incubated at 37°C in a 5% CO_2_ incubator for 48 h. The breadth of the initial scratched gap and the residual scratched gap was measured, as images of migrating cells were sequentially taken at 0 h, 12 h, 24 h, and 48 h after scratching.

### Drug resistance

Cell viability was assessed by an MTT assay after oxaliplatin (L-OHP) administration. LGR5-transfected cells and mock-transfected cells were seeded in a 96-well plate at a density of 1×10^4^ cells/well. After 24 hours of incubation at 37°C with 5% CO_2_, L-OHP was added at concentrations of 2.5, 5, 10, 20 and 40 μM/L. The cell viability was evaluated after 48 hours of L-OHP treatment by MTT assay.

### Xenograft tumors in nude mice

Female BALB/c nude mice (4–5 weeks of age, 18–20 g), purchased from BEIJING HFK Biological CO., LTD (Beijing, China) and housed in the Laboratory Animal Center of Hebei University Health Sciences Center (Baoding, China), were used to examine tumorigenicity. For xenograft studies, an equal number (2x10^3^, 2x10^4^, 2x10^5^, and 2x10^6^) of freshly dissociated tumor sphere cells or control adherent cells was injected subcutaneously into each mouse (n = 3 per group). The tumor volumes were determined according to the following formula: v (mm^3^) = length x width^2^/2. The tumor diameters were measured every two days using vernier calipers. After 5 weeks, the mice were sacrificed, and the tumors were removed and weighed. The tumor tissues were sectioned at 5 μm for H&E staining. All experimental procedures and protocols were approved by the Animal Welfare and Ethical Committee of Hebei University.

### Flow cytometry

The distribution of LGR5, CD44 and CD54 on human gastric cancer cells was assessed by flow cytometry using a PE-conjugated mouse anti-human LGR5 mAb (OriGene, Rockville, MD), FITC-conjugated rat anti-human CD44 antibody and APC-conjugated mouse anti-human CD54 antibody (eBioscience, US). The cells were washed twice with PBS and then labeled with a fluorescence-conjugated antibody by a 15-min incubation on ice, followed by three additional washes. The flow cytometry analysis was performed on a BD FACSCalibur ^™^ instrument (Becton, Dickinson and Company, US).

### Immunofluorescence staining

Mechanically dissociated tumor sphere-forming cells or adherent cells were fixed in 4% paraformaldehyde (Solarbio) for 30 min at room temperature. The cells were washed three times with PBS, incubated for 1 hour in 1% BSA blocking buffer containing 10% goat serum, and then incubated with an anti-LGR5 mouse monoclonal antibody (TA503316, OriGene, Rockville, MD) at a 1:400 dilution overnight at 4°C. After three 10-min washes with PBS, the cells were incubated with the secondary antibody, a FITC-conjugated goat anti-Mouse IgG antibody (SA00003-1, Proteintech, USA) at 1:100 dilution in 1% BSA blocking buffer, for 30 min at 37°C. After three 10-min washes with PBS, the cells were incubated with 0.1 μg/ml DAPI (Sigma, USA) at room temperature for 10 min. The cells were then washed with PBS three times for 5 min each and observed using an Olympus fluorescence microscope.

## Results

### Cancer tissues are not useful starting materials to detect stemness and EMT core factors

**Fig 1:**
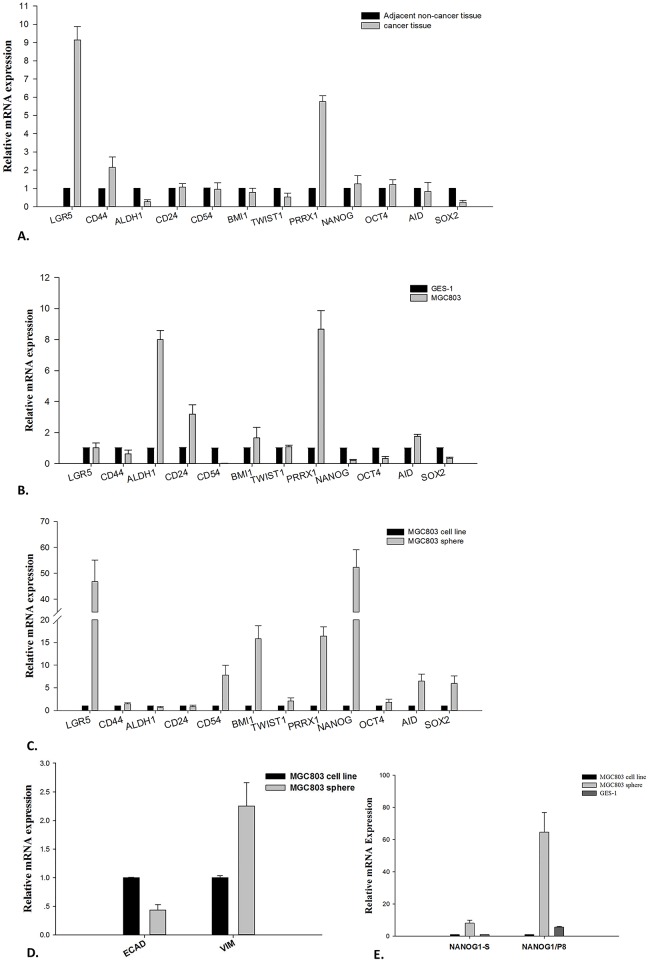
Detection of gene expression profiles by qPCR. **A**. Gastric cancer tissues versus adjacent normal tissues. **B**. MGC803 (a poorly differentiated cancer cell line) adherent cells versus GES-1 (a gastric epithelial cell line) adherent cells. **C**. MGC803 sphere cells versus MGC803 adherent cells. **D**. Expression of E-cadherin and vimentin in sphere cells versus parental adherent cells. **ECAD** stands for E-cadherin; **VIM** stands for vimentin. **E**. Expression of NANOG1-S and NANOG1/P8 in sphere cells versus parental adherent cells. **NANOG1-S** stands for NANOG1-specific primers and **NANOG1/P8** stands for NANOG1 and NANOGP8 shared primers.

We collected fresh cancer tissues and adjacent normal tissues from 9 patients with metastasis who had just undergone a gastrectomy procedure, and the cancer and normal tissues were separately pooled for total RNA extraction. A transcript analysis was performed using qPCR with primers against a set of 12 genes, including 5 putative CSC markers, *LGR5*, *CD44*, *ALDH1*, *CD24*, and *CD54*; 4 stemness regulators, *NANOG*, *OCT4*, *SOX2* and *AICDA*; and 3 EMT inducers, *PRRX1*, *TWIST1*, and *BMI1*. The qPCR results showed a significant up-regulation of only *LGR5*, *CD44* and *PRRX1* (>2-fold), and not the other genes, in cancer tissues (Fig 1A).

### Cancer cell lines are also not proper starting materials to detect stemness and EMT properties

Since the qPCR analysis of gastric cancer tissues only detected up-regulated expression of *LGR5*, *CD44* and *PRRX1* among the putative CSC markers, stemness regulators and EMT inducers, we next tried to use GC cell lines *in vitro* to confirm this result. We conducted a qPCR analysis of the same set of genes using the poorly differentiated gastric adenocarcinoma cell line MGC803 as the test material and GES-1, a normal gastric epithelial cell line, as a reference. The results showed up-regulated expression of only *ALDH1*, *CD24*, and *PRRX1* (up to >2-fold), but not the other genes, in MGC803 cells (Fig 1B).

### Sphere cells are the best materials to detect the expression profile of stemness and EMT core genes

**Fig 2:**
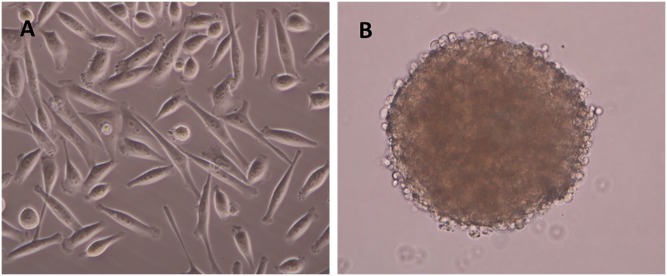
Microscopic observation of sphere cells and adherent cells. **A**. MGC803 adherent cells. **B**. MGC803 sphere cells. All the images are 400× magnified.

Considering that the MGC803 and GES-1 cell lines represent heterogeneous cells and that CSC cells make up only a tiny portion of the cell population, we, therefore, speculated that the enrichment of CSC-like cells should be the first step before any further isolation efforts. To prove our hypothesis, we conducted sphere cell culture with MGC803 cells (Fig 2A and 2B) and then used qPCR to detect the relative mRNA expression in sphere cells versus the parental adherent cells. Not surprisingly, the MGC803 sphere cells showed a significant up-regulation of not only the putative CSC markers *LGR5* and *CD54* but also the stemness regulators *NANOG*, *OCT4*, *SOX2*, and *AID* and the EMT inducers *TWIST1*, *PRRX1*, and *BMI1* (Fig 1C). Among these genes, *LGR5* and *NANOG* showed the highest expression in sphere cells; the former was increased more than 45-fold, and the latter more than 50-fold. This result clearly demonstrates that only sphere cells can be used to detect CSC gene expression profiles sensitively, while cancer tissues and cancer cell lines cannot.

### Sphere cells showed down-regulated E-cadherin and up-regulated vimentin expression

The above qPCR experiment only detected EMT-associated transcription factors but did not detect the structural proteins that endow the cells with the EMT phenotype. Therefore, we conducted a qPCR analysis to detect the expression profiles of E-cadherin and vimentin, which are sensitive indicators of cells going through the EMT process [24, 25]. The results showed that E-cadherin is significantly down-regulated (-2.23-fold) and vimentin is significantly up-regulated (2.25-fold; see Fig 1D) in sphere cells. This observation clearly shows that sphere cells acquire certain properties of mesenchymal cells through the EMT process.

### Most of the *NANOG* expression is contributed by the retrogene *NANOGP8*

Our qPCR results showed that the expression of *NANOG* is highly up-regulated in sphere cells; however, *NANOG* represents a family of 11 genes, including 1 canonical gene (*NANOG1*), 1 duplicated gene (*NANOG2*), 1 retrogene (*NANOGP8*), and 8 pseudogenes, all of which show a high sequence similarity to each other, but only the first 3 of which are known to be expressed [26]. It has been reported that *NANOG1* is primarily expressed in embryonic cells, and the retrogene *NANOGP8* is mainly expressed in cancer cells. Therefore, we wanted to understand the differential expression of *NANOG1* and *NANOGP8* in cancer sphere cells in a quantitative way. We conducted a qPCR analysis using *NANOG1*-specific primers and *NANOG1/NANOGP8* (*NANOG1/P8*) shared primers (Table 1) because the 5’ end of the *NANOG1* transcript contains a 22-bp stretch of unique sequence, while most of the sequence in the remaining coding region is identical for both *NANOG1* and *NANOGP8* [27]. The qPCR results showed that the *NANOG1*-specific expression is up-regulated about 5-fold in sphere cells, while *NANOG1/P8* expression is up-regulated more than 50-fold in sphere cells (Fig 1E). This result indicates that most of the *NANOG* expression in sphere cells actually originates from *NANOGP8*. In addition, we compared the expression of *NANOG1* and *NANOG1/P8* in the different CSC sources. The results showed a trend of increasing *NANOG1* expression from sphere cells to adherent cancer cells to GES-1 cells, and a trend of increasing *NANOG/P8* expression from sphere cells to GES-1 cells to adherent cancer cells (Fig 1E). Finally, we analyzed the *NANOG* expression in other gastric cell lines, including MKN45, SGC-7901, and HGC27, and the corresponding sphere cells. We observed the highest *NANOG/P8* expression in sphere cells derived from poorly differentiated cancer cell lines (MGC-803, MKN45) and lower *NANOGP8* expression in moderately differentiated cell lines (SGC-7901). However, the *NANOG1* expression was significantly higher than the *NANOG1/P8* expression in undifferentiated cells (HGC27) and sphere cells (data not shown). This result may reflect the fact that undifferentiated cells have more characteristics of embryonic cells.

NANOG1-S stands for *NANOG1*-specific primers; NANOG1/P8 stands for *NANOG1* and *NANOGP8* shared primers; ECAD stands for E-cadherin; and VIM stands for vimentin.

### Detection of LGR5 and NANOG proteins in sphere cells and their parental adherent cells

**Fig 3:**
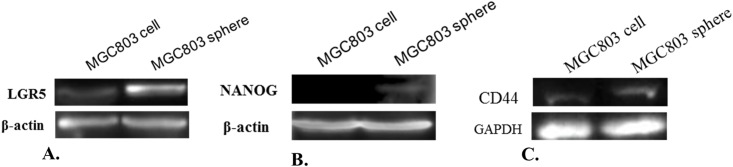
Detection of LGR5, NANOG and CD44 proteins in MGC803 sphere cells and adherent cells.

To make sure our qPCR results were consistent with protein expression, we detected LGR5 and NANOG protein expression by western blot analysis, and the results demonstrated that high mRNA expression was correlated with high protein expression (Fig 3A and 3B). Both LGR5 and NANOG proteins were significantly up-regulation in sphere cells; however, we are not sure from which gene the NANOG protein originates, NANOG1 or NANOGP8, because we do not have a NANOGP8-specific antibody. In addition, we detected CD44 protein levels. The result shows an equal expression level in both adherent cells and sphere cells (Fig 3C), which is consistent with our qPCR result (Fig 1C). These results demonstrate that sphere cells exhibit increased expression of the LGR5 and NANOG proteins, but not CD44, and that NANOG may be responsible for CSC properties.

### LGR5 over-expression promotes gastric cancer cell proliferation and sphere cell growth

**Fig 4:**
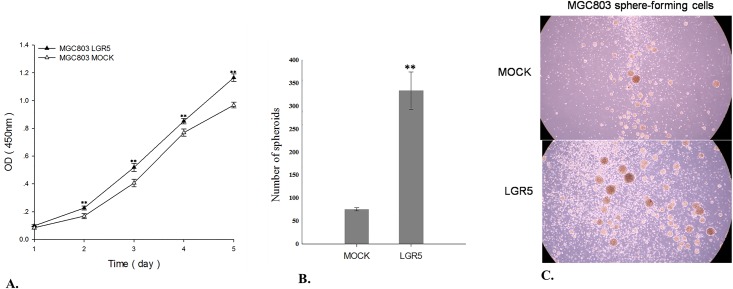
Result of cell proliferation and sphere cell growth assays. **A**. Cell proliferation assay results for LGR5-transfected MGC803 cells and the mock-transfected MGC803 cells. **B**. Sphere cell growth assay results for LGR5-transfected MGC803 cells and the mock-transfected MGC803 cells. **C**. Images of the sphere cell growth in LGR5-transfected and mock-transfected conditions. **p<0.01.

To explore if LGR5 can enhance cancer cell expansion and survival and reduce apoptosis, we conducted a cell proliferation assay with LGR5-transfected MGC803 cells and mock-transfected control cells. The result showed that the over-expression of LGR5 in MGC803 cells promotes gastric cancer cell proliferation significantly (*p*<0.01) (Fig 4A). To further confirm the LGR5 association with the stemness of CSCs, we compared the sphere cell growth capability of LGR5-transfected and mock-transfected MGC803 cells. The result showed that the sphere numbers derived from LGR5-transfected cells were 4 times higher than those from mock-transfected control cells (Fig 4B and 4C). This result clearly demonstrates that the aberrant expression of LGR5 is significantly associated with cell stemness.

### LGR5 over-expression promotes gastric cancer migration

**Fig 5:**
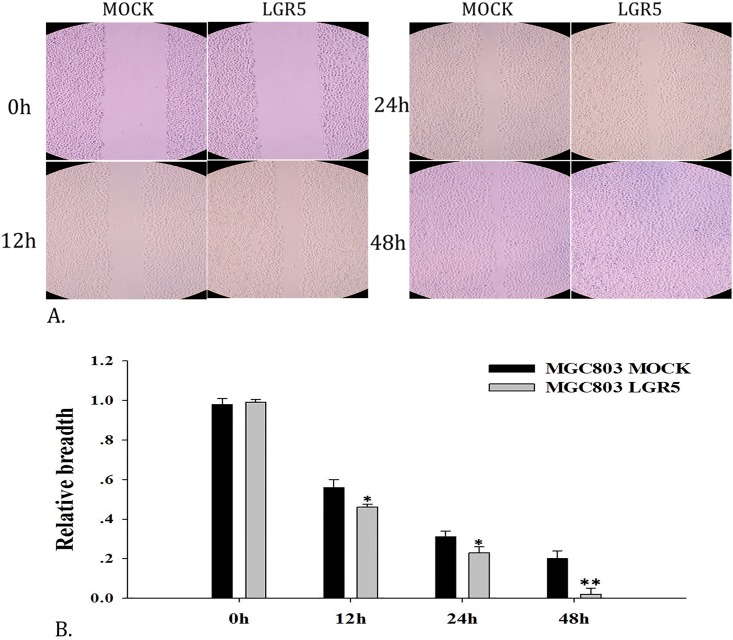
Cell migration analysis by wound-healing assays. MGC803 cells transfected with LGR5 and mock-transfected cells were compared. **A**. The cell wounds were visualized at 0 h, 12 h, 24 h, and 48 h; **B**. Migration analysis at different time points. *p<0.05; **p<0.01.

To further confirm that LGR5 can promote cancer cell migration and even metastasis, we performed a cell migration assay with LGR5-transfected MGC803 cells and the mock-transfected control cells. The result demonstrated that the over-expression of LGR5 in MGC803 cells significantly enhance gastric cancer cell migration (*p*<0.01), which indicates that LGR5 is associated with cell migration and invasiveness (Fig 5A and 5B).

### LGR5 over-expression induces oxaliplatin (L-OHP) resistance in MGC803 cells

**Fig 6:**
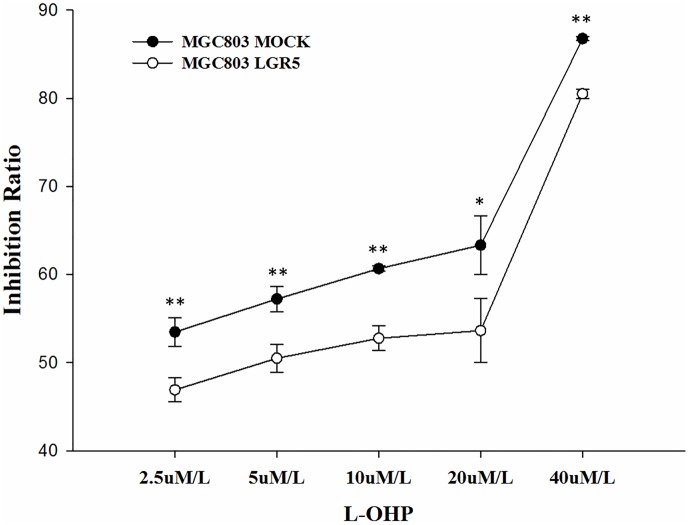
Drug resistance analysis by MTT assay. MGC803 cells transfected with LGR5 and mock-transfected cells were compared. The black line with solid circles represents MGC803 mock-transfected cells, and the gray line with hollow circles represents LGR5-over-expressing cells. *p<0.05; **p<0.01.

To explore if LGR5 is associated with chemotherapy resistance in MGC803 cells, we conducted an MTT assay to evaluate the viability of LGR5-transfected and mock-transfected cells after treatment with **oxaliplatin (L-OHP)**, a platinum-based antineoplastic agent used as chemotherapy in cancer clinics. Forty-eight hours after the administration of L-OHP, LGR5-transfected cells showed significant drug resistance (15% to 20%) compared to mock-transfected cells to L-OHP concentrations of 2.5 μM/L, 5 μM/L, 10 μM/L, 20 μM/L and 40 μM/L (Fig 6).

### Tumorigenicity of sphere cells in NOD/SCID mice

**Fig 7:**
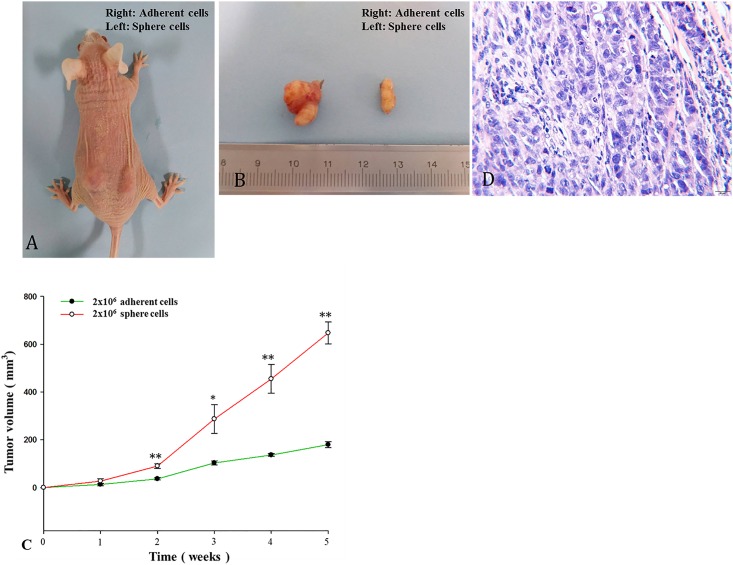
Tumor growth after subcutaneous inoculation in nude mice. **A**. Tumor growth in nude mice after injection of MGC803 tumor sphere cells (2X10^6^) into the left rear flank of mice and the parental adherent cells (2X10^6^) into the right rear flank of mice. **B**. Representative xenograft tumors are shown. **C**. Size comparison of subcutaneous tumors following the injection of an equal number of MGC803 tumor sphere cells and MGC803 adherent cells (2X10^6^). *p<0.05; **p<0.01. **D**. H&E staining analysis of xenograft tumors derived from MGC803 sphere cells. The scale bars = 20 μm.

To further confirm that sphere cells represent enriched CSCs with enhanced tumor initiation potential *in vivo*, we conducted tumor xenograft experiments in NOD/SCID mice. The result showed that sphere cells are at least 10-fold more efficient at tumor initiation than the parental adherent cells (Table 2). With the same number of cells inoculated into mice, the sphere cells induced much larger tumors than the adherent cells (Fig 7A–7C). A histological examination demonstrated that the xenograft tumors in mice possess the same histological heterogeneity as those in human primary gastric cancer (Fig 7D).

**Table 2:** Tumor initiation rate in nude mice by sphere and adherent cells.

Cells injected	Adherent cells	Sphere cells
2x10^3^	0/3	0/3
2x10^4^	1/3	0/3
2x10^5^	3/3	0/3
2x10^6^	3/3	3/3

### Flow cytometry to detect cell sub-populations with different markers

**Fig 8:**
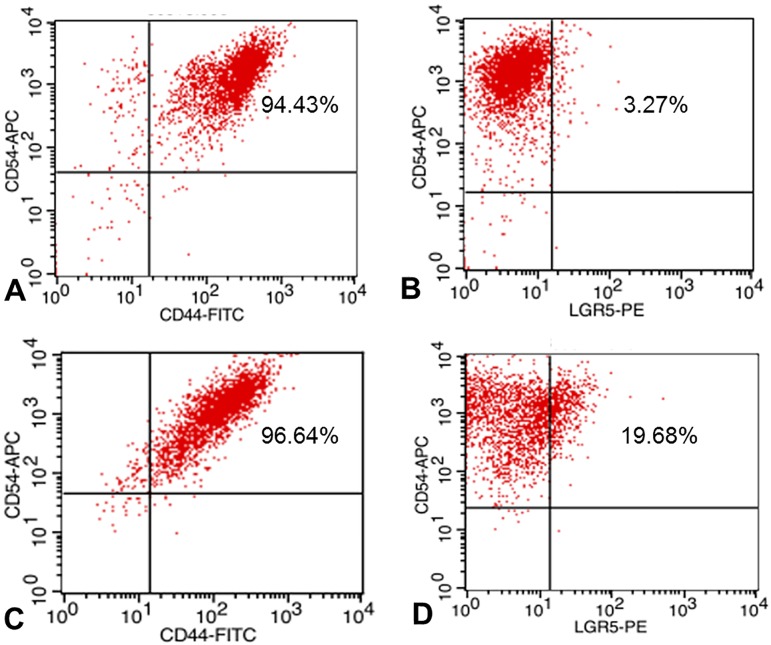
Flow cytometry analysis of cell subpopulations with different markers. **A**. CD54+/CD44+ cells in MGC803 adherent cells; **B**. LGR5+/CD54+ cells in MGC803 adherent cells; **C**. CD54+/CD44+ cells in MGC803 sphere cells; and **D**. LGR5+/CD54+ cells in MGC803 sphere cells.

To understand cell types in sphere-forming cells, we conducted flow cytometry to sort cell sub-populations with different markers such as LGR5, CD54, and CD44. The results showed that more than 94% of the parental adherent cells are CD54+/CD44+ and more than 96% of sphere cells are CD54+/CD44+ (Fig 8A and 8C). By contrast, only approximately 3% of the parental adherent MGC803 cells are LGR5+/CD54+, while approximately 20% of sphere cells are LGR5+/CD54+ (Fig 8B and 8D). Obviously, the proportion of LGR5+ cells is significantly increased in sphere cells, while the proportion of CD54+/CD44+ cells is the same in both adherent cells and sphere cells. We believe that the LGR5-expressing fraction of the CD54+ cells may represent the gastric CSCs.

### Immunostaining to detect LGR5+ in adherent and sphere-forming cells

**Fig 9:**
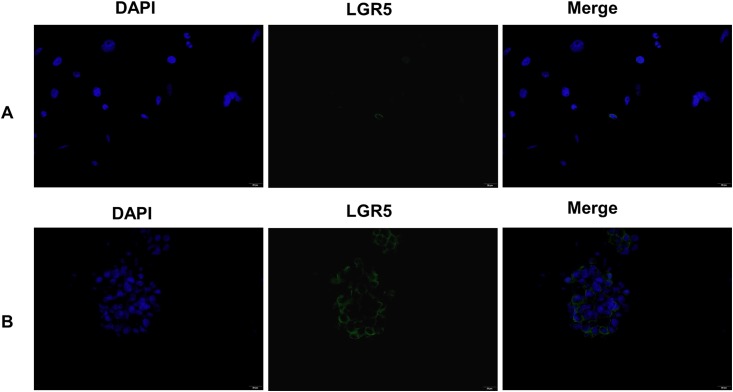
Immunofluorescence staining of LGR5+ cells. **A**. LGR5+ cells in MGC-803 adherent cells; **B**. LGR5+ cells in MGC803 sphere cells. All the images are 400× magnified.

To confirm our flow cytometry results, we performed immunofluorescent staining of sphere cells and the parental adherent cells with an LGR5-specific mAb. The results showed that few LGR5+ cells exist in MGC803 adherent cells (Fig 9A), while many more LGR5+ cells exist in sphere cells (Fig 9B). This observation is consistent with our flow cytometry analysis.

## Discussion

Our previous immunohistochemistry results showed that LGR5+ cells are located at the very base of the gastric normal gland [12]. Our present data showed that *LGR5* expression is highly up-regulated in GC tissues and sphere cells derived from GC cell lines, but *LGR5* association with stemness and EMT signature genes can be detected only in sphere cells. These data strongly suggest that LGR5 is a potential dual marker for both normal adult stem cells and CSCs. It also demonstrates that sphere cells are the best starting materials for studying CSC properties. Several cell-surface proteins were reported to be dual markers as well. For example, CD133 was reported as a CSC marker in brain [16], lung [28], pancreatic [29] and prostate cancer [30], while it was also reported to be a reliable stem cell marker in normal adult tissues such as brain [31], prostate [32] and kidney [33].

In addition to LGR5, several other proteins have been reported as putative CSC markers in GC, including CD44 [12], ALDH1 [13], CD44/CD24 [15] and CD44+/CD54+ [14]. Our qPCR results showed that of these markers, only *CD54* is up-regulated in sphere cells. Our flow cytometry analysis showed that the proportion of CD54+ cells in sphere cells is about the same as that in the parental adherent cells, while a remarkable increase in LGR5+ cells was observed in sphere cells (~20%) compared to that in the parental adherent cells (~3%, Fig 8). Immunofluorescence staining results showed a significant increase of LGR5+ cells in sphere cells but few in the parental adherent cells (Fig 9). Furthermore, LGR5-transfected GC cells showed significantly enhanced cell proliferation (Fig 4A), sphere cell growth (Fig 4B and 4C), cell migration (Fig 5), and drug resistance (Fig 6). Taken together, our data strongly demonstrate that the LGR5+ fraction of CD54+ cells is a possible CSC population, and CD54+ alone or CD54+/CD44+ cells are not.

Sphere cell culture is a newly established technology for growing stem-like cells, though there are some arguments against it [16]. Many groups have obtained sphere cells from solid tumors of cancers such as breast, colon, lung, pancreas, and ovarian [34,18,29,35,36], and all of the sphere cells showed stem cell characteristics, including *in vivo* tumorigenicity with NOD/SCID mice. We characterized LGR5 and its association with 5 putative CSC markers, 4 stemness regulators, and 3 EMT inducers in three cell sources, i.e., gastric cancer tissues, gastric cancer cell lines and sphere cells. The results demonstrated that LGR5, stemness and EMT markers are simultaneously up-regulated only in sphere cells (Fig 1A–1C). We also detected E-cadherin and vimentin expression. The expression of E-cadherin is significantly down-regulated in sphere cells, and the expression of vimentin is significantly up-regulated in sphere cells, which confirmed the close association of sphere cells with EMT. There are many reasons underlying this observation, and we believe the major reason is that CSCs represent a tiny portion of the tumor mass, so their signal is masked by overwhelming noise from the heterogeneous cells in the tumor cell population, while sphere culture enriches the CSCs to a threshold that can effectively suppress the non-CSC noise. Overall, our data clearly indicate that CSC enrichment by sphere cell culture is a key step for the efficient study of CSCs.

To further confirm that the sphere cells are CSC-like cells, we conducted xenograft tumor experiments *in vivo*. The results clearly showed that sphere cells are significantly more efficient (at least 10-fold) at tumor initiation *in vivo* than the parental adherent cells (Fig 7). This observation strongly argues that sphere cell culture can enrich CSCs and that CSCs are responsible for tumor initiation. This observation is consistent with our results of gene expression, cell proliferation, cell migration, flow cytometry and immunofluorescent staining assays.

It has been increasingly recognized that cancer drug resistance is closely associated with EMT and CSCs in many cancers [37, 38, 39]. Our study is the first to show the association of drug resistance with EMT and CSCs in gastric cancer cells. We demonstrated that LGR5 is not only closely associated with CSCs and EMT, it is also significantly associated with drug resistance. The fact that LGR5-overexpressing cells show high resistance to L-OHP at various concentrations strongly argues that CSCs and EMT are intimately associated with drug resistance in gastric cancer cells. It suggests that a drug targeting the LGR5-expressing tumor cells, i.e., the CSCs, could be an ideal way to treat gastric cancer or prevent relapse and metastasis.

Our results demonstrated that *NANOG* expression is up-regulated more than 50-fold in sphere cells, which suggests that *NANOG* may play an important role in maintaining CSC properties. Using *NANOG1*-specific primers, we found that though both *NANOG1* and *NANOGP8* are up-regulated in sphere cells, most of the *NANOG* expression in sphere cells is actually contributed by *NANOGP8* (Fig 1E). *NANOGP8* is a species-specific retrogene in humans [26]. Its high expression in tumor CSCs suggests it plays certain roles in stemness acquisition and tumorigenesis. It is believed that *NANOGP8* expression is low in embryonic cells and high in cancer cells, but we observed that the *NANOGP8* expression was higher in the normal epithelial cell line GES-1 than in the GC cancer cell line MGC803. Since the GES-1 cell line was established from fetal stomach tissue and represents normal gastric epithelial cells, our result actually conflicts with the above hypothesis. These cells may be an exception, or our result may represent some unknown fact. Recently, it was reported that NANOG plays an essential role in adult cell reprogramming, malignant cell transformation and cancer initiation, while OCT4 and SOX2 are dispensable [40, 41, 42, 43, 44]; however, these reports did not distinguish between NANOG1 and NANOGP8. Nevertheless, these reports are consistent with our observation that the expression of *NANOG1/P8* in sphere cells is significantly higher than that of *OCT4* and *SOX2*. Evidence shows that NANOG expression is regulated by AICDA, which was originally known to be responsible for antibody diversity. Experiments using siRNA-mediated knockdown demonstrated that AICDA activity was required to demethylate the promoters of NANOG and OCT4, which in turn initiate pluripotency in mouse induced stem cells [45]. It has also been reported that NANOG can serve as an EMT inducer. Experiments with skin epithelia showed that NANOG can bind to the promoters of and directly activate the EMT-associated genes TWIST1 and PRRX1, and twist1 can repress E-cadherin, which confers EMT features on cells [46, 47]. This report is consistent with our observation that up-regulated *AICDA* and *NANOG* are associated with the EMT factors *TWIST1*, *PRRX1* and *BMI1* and the stemness factors *OCT4* and *SOX2*, which strongly suggests that stemness and EMT are closely coupled together in CSCs. In future studies, carefully distinguishing between *NANOG1 and NANOGP8* should be a key focus.

PRRX1 is an exceptional EMT regulator because it is up-regulated in all cell sources, including gastric cancer tissues, gastric cancer cell lines and sphere cells. PRRX1 is a homeodomain transcription factor and a newly discovered EMT inducer. It was initially thought to be a biomarker associated with patient survival and was believed to decrease metastasis by uncoupling EMT and stemness [48]. Recently, it was reported that up-regulated PRRX1 is closely correlated with metastasis and poor prognosis in colorectal and pancreatic cancers [49, 50] and enhances invasiveness in glioblastoma cell lines [51]. These observations are in line with our results that *PRRX1* is highly co-expressed with *BMI1* and *TWIST1*. TWIST1 is a bHLH transcription factor involved in EMT, while BMI1 is a chromatin remodeling factor involved in the cell stemness of normal and cancer tissues. Our previous data showed that BMI1 is actually co-expressed with LGR5 [11]. It was previously known that BMI1 promotes cancer resistance to chemotherapy and radiotherapy, but its association with EMT was not known until a recent report demonstrating that BMI1 is directly activated by TWIST1; in turn, these proteins cooperatively promote EMT and the tumor-initiating capability of cancer cells [52]. The fact that PRRX1 promotes EMT through the Wnt/β-catenin pathway in gastric cancer [53] and that LGR5 is a co-receptor of the Wnt/β-catenin pathway involved in tumorigenesis clearly suggests an intimate association between PRRX1, LGR5 and EMT. The significant PRRX1 up-regulation observed in a variety of gastric cancer cell sources could explain the high malignancy and poor outcomes of gastric adenocarcinoma because PRRX1 is a strong driver of both EMT and metastasis.

To further confirm our hypothesis, we measured the gene expression of E-cadherin and vimentin, which are indicators of the EMT process in cells. It has been well accepted that down-regulated E-cadherin and up-regulated vimentin expression in the EMT process are unique features of the metastatic property in cancer cells. Loss of E-cadherin is a key initial step in EMT, which is mediated by the binding of EMT transcription factors such as TWIST1 to E-boxes present in the E-cadherin promoter [47], while vimentin is a Wnt activity-targeted gene in mesenchymal cells involved in metastasis [54]. Our qPCR results show that in sphere cells, the expression of E-cadherin is significantly down-regulated while the expression of vimentin is significantly up-regulated (Fig 1D). This result supports the observations that *LGR5* may activate vimentin via enhanced Wnt activity and repress E-cadherin via up-regulated *TWIST1* and *BMI1* expression (Fig 1C).

In summary, we employed a variety of techniques to study the expression of LGR5 and its correlation with stemness and EMT core factors. Our results show that *LGR5* expression is highly up-regulated in sphere cells and closely associated with the stemness regulators *NANOG*, OCT4, *SOX2*, *AICDA* and the EMT inducers *TWIST1*, *PRRX1* and *BMI1*. Flow cytometry analysis showed that LGR5+ cells are increased significantly in sphere cells, but the level of CD54+/CD44+ cells is the same in both sphere cells and parental adherent cells. The GC cells transfected with *LGR5* showed significantly enhanced cell proliferation, sphere cell growth, cancer cell migration, and drug resistance. *LGR5* may activate *TWIST1*, which in turn down-regulates E-cadherin, and *LGR5* may also up-regulate vimentin via enhanced Wnt-signaling activity. Finally, our xenograft tumor experiment in nude mice demonstrates that sphere cells are at least 10 times more efficient at tumor initiation than the parental adherent cells. We concluded that LGR5 is closely associated with stemness and EMT properties and that the LGR5-expressing fraction of CD54+ cells represents the gastric CSCs. Our results also demonstrate that sphere cells are the best starting materials to characterize CSCs and to avoid misleading data. To the best of our knowledge, this is the first report demonstrating the stable and simultaneous expression of LGR5 and stemness/EMT signature genes in GC sphere cells and that NANOG expression mainly comes from the retrogene *NANOGP8*, all of which are associated with drug resistance. We believe that our data will facilitate the study of gastric CSCs and the development anti-cancer drugs in the future.

## Supporting Information

S1 Fig LGR5 proteins in MGC803 sphere cells and adherent cells. (TIF)

S2 Fig NANOG proteins in MGC803 sphere cells and adherent cells. (TIF)

S3 Fig CD44 proteins in MGC803 sphere cells and adherent cells. (TIF)
