# Supplementary figures and images for: LGR5 Is a Gastric Cancer Stem Cell Marker Associated with Stemness and the EMT Signature Genes NANOG, NANOGP8, PRRX1, TWIST1, and BMI1
Source: PLoS One. 2016 Dec 29;11(12):e0168904. doi: 10.1371/journal.pone.0168904 (PMC5199039; doi:10.1371/journal.pone.0168904)

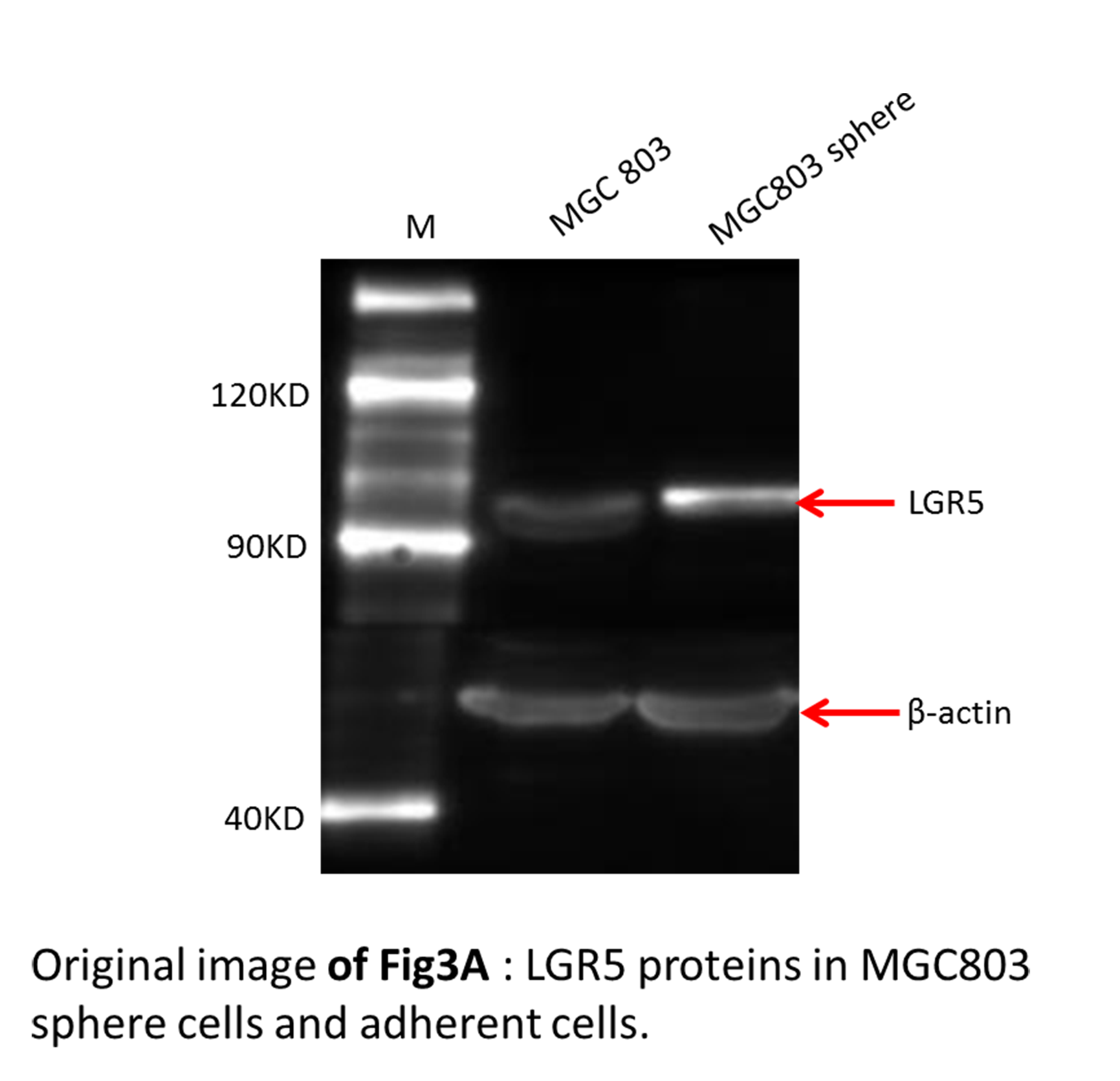

Supplement: S1 Fig — (TIF) [file pone.0168904.s001.tif]

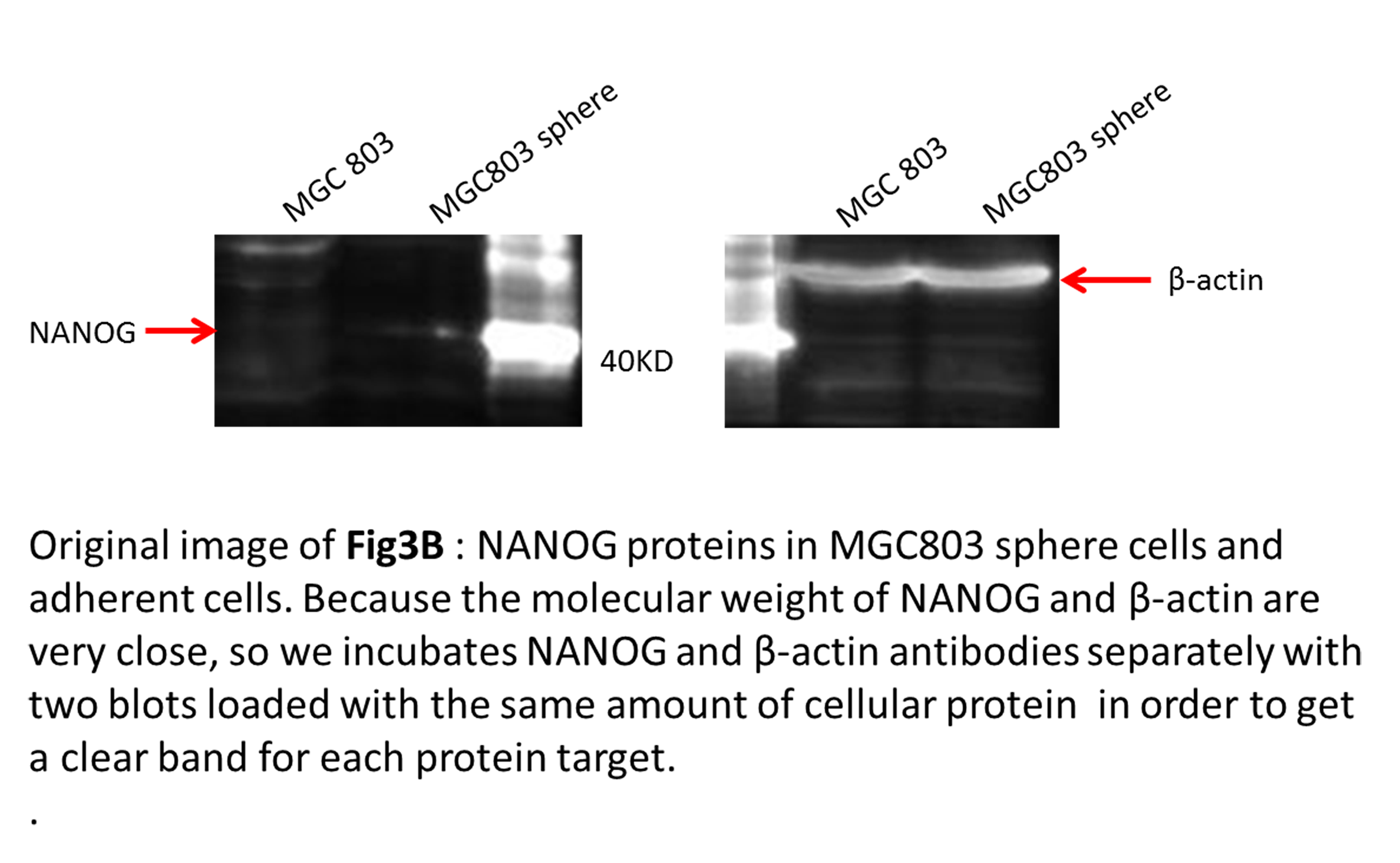

Supplement: S2 Fig — (TIF) [file pone.0168904.s002.tif]

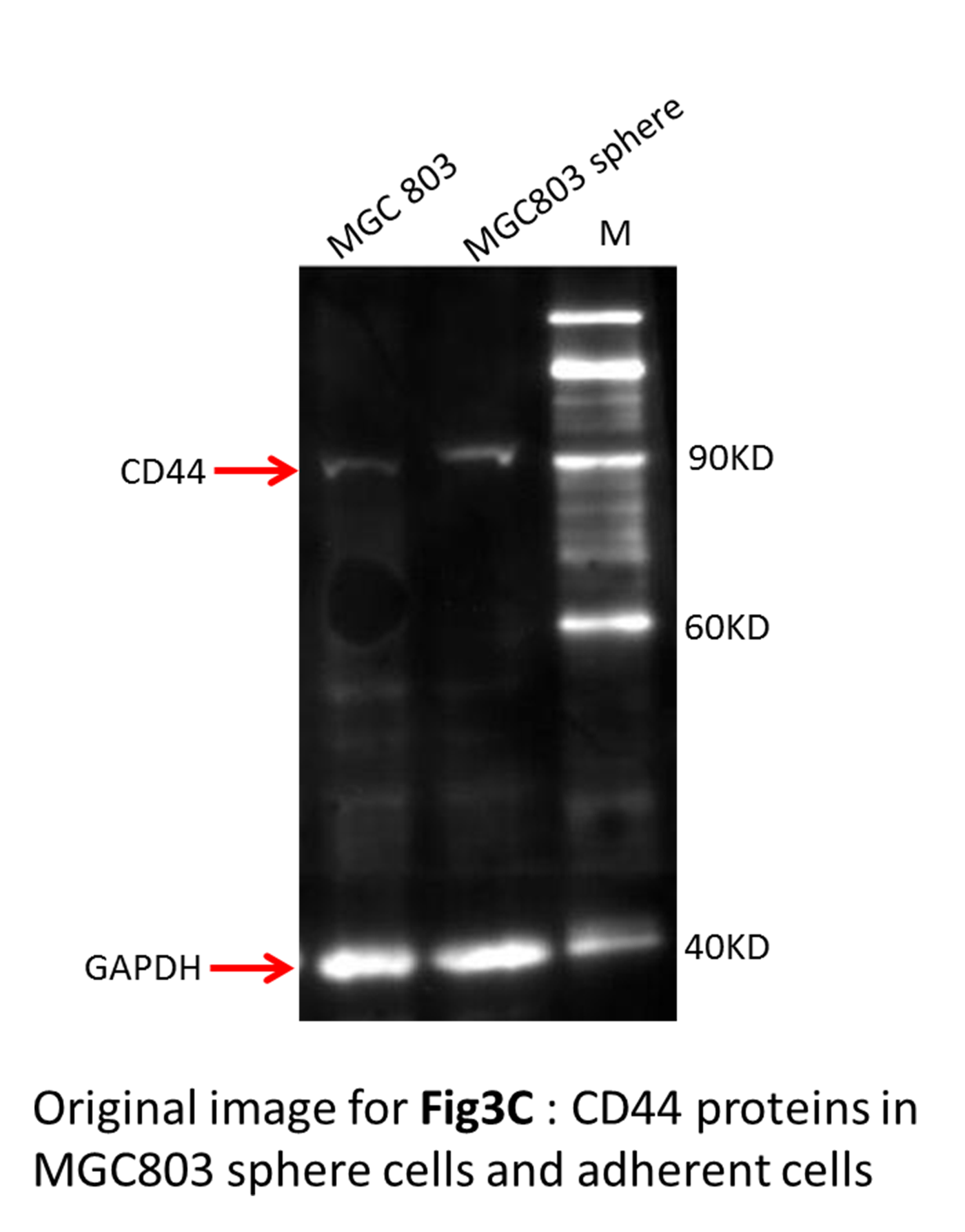

Supplement: S3 Fig — (TIF) [file pone.0168904.s003.tif]
